# Range Expansion and Environmental Determinants of the Invasive Freshwater Leech *Helobdella europaea* (Hirudinea: Glossiphoniidae) in Morocco

**DOI:** 10.3390/biology15151228

**Published:** 2026-07-23

**Authors:** Fouzi Abdelkhaleq Taybi, Serge Utevsky, Youness Mabrouki

**Affiliations:** 1 Synergy Lab, Higher School of Education and Training, Mohammed I University, BP-410, Oujda 60000, Morocco; a.taybi@ump.ac.ma; 2 Department of Zoology and Animal Ecology, V. N. Karazin Kharkiv National University, Maidan Svobody, 4, 61022 Kharkiv, Ukraine; serge.utevsky@karazin.ua; 3 Biotechnology, Conservation and Valorisation of Bioresources Laboratory (BCVNR), Faculty of Sciences Dhar El Mehraz, Sidi Mohamed Ben Abdellah University, Fez 30000, Morocco

**Keywords:** biological invasion, invasive leech, freshwater biodiversity, North Africa, biomonitoring, range expansion

## Abstract

**Simple Summary:**

Invasive species can threaten native biodiversity and disrupt ecosystems, making it important to monitor their spread and understand the factors that help them establish in new environments. This study examined the distribution of *Helobdella europaea*, a non-native freshwater leech, across northern Morocco between 2014 and 2024. Surveys were carried out in a variety of aquatic habitats, including protected wetlands and major river basins. The results showed that this species has expanded its range considerably since it was first recorded in Morocco. It was found in many different habitat types, including environmentally degraded sites, demonstrating a high ability to tolerate diverse environmental conditions. Among the factors studied, water flow and salt content were the main environmental conditions limiting its occurrence and abundance. The findings indicate that *H. europaea* has a strong potential to continue spreading throughout Moroccan freshwater ecosystems. Improving our understanding of the environmental conditions that influence its distribution will help support biodiversity conservation and the management of invasive species, particularly as hydrological alterations and climate change may create new opportunities for its expansion in the future.

**Abstract:**

Invasive species represent a major threat to global biodiversity, highlighting the need for continuous monitoring and research to better understand their ecology, distribution, and invasion dynamics. Among freshwater leeches (Hirudinea), *Helobdella europaea* is considered one of the most successful and widespread invasive taxa. This study provides an updated assessment of the distribution of *H. europaea* in Morocco and identifies the main environmental factors influencing its occurrence and spread across the country. Field surveys were conducted between 2014 and 2024 in northern Morocco, with particular focus on protected wetlands, including Ramsar sites, and major biogeographical barriers such as the Middle Atlas Mountains and the Sebou and Moulouya river basins. Specimens were collected using Surber samplers (20 × 25 cm, 400 μm mesh), while physicochemical water parameters and other abiotic variables were simultaneously recorded. Since its first detection in Morocco in 2014, *H. europaea* has significantly expanded its geographic range. The species exhibited broad ecological tolerance, occurring in a wide variety of habitats, including environmentally degraded sites, and enduring substantial variation in water conditions. Among the environmental variables examined, water velocity and salinity were identified as the primary factors constraining the species’ distribution and abundance. Nevertheless, additional studies are required to better predict the future expansion dynamics of *H. europaea*, particularly under ongoing hydrological alterations and climate change scenarios.

## 1. Introduction

The introduction of non-native species is widely recognized as one of the primary drivers of biodiversity loss in aquatic ecosystems [1,2,3]. In recent decades, biological invasions have increasingly been regarded as a major component of global environmental change, particularly in freshwater and marine environments [4]. This phenomenon has been further intensified by the rapid expansion of global trade and transportation networks, which have substantially increased both the frequency and geographical scale of species introductions worldwide [5]. In addition to their ecological consequences, invasive species may also pose serious threats to public health [6] and generate considerable economic costs at both local and global scales [7].

Among aquatic invasive taxa, non-native freshwater leeches can have profound effects on the biodiversity and functioning of the ecosystems they invade [8]. Several leech species have emerged as particularly successful invaders in recent decades, some exhibiting remarkable dispersal capacities, including transcontinental and transoceanic expansion [9,10]. Beyond their ecological impacts, freshwater leeches may also serve as vectors of parasites and pathogens, potentially facilitating disease transmission through their parasitic interactions with a broad range of vertebrate hosts [11,12,13].

A good example of a successful biological invasion within the Hirudinea class is *Helobdella europaea* (Kutschera, 1987), which is considered an invasive species with a complicated taxonomic history. It has been described more than once in the last 40 years [14]. Although the type locality of *H. europaea* is in Germany [15,16]—it was once thought to be native to Europe—the origin of *H. europaea* is from the Nearctic and native to North America [17]. It is believed that the original description was made after the species had already been introduced to Europe [18], as has been observed with other North American invaders [19,20].

The number of recorded alien species in Morocco far exceeds the number of studies evaluating their ecological impacts on freshwater ecosystems [21]. In North Africa, *H. europaea* has so far been recorded only in Morocco, where it was first detected in 2014, and at just two locations [22]. The primary aim of this study is to update the available data on *H. europaea* in Morocco and to identify the main environmental factors influencing its distribution. A second objective is to confirm the species’ ecological plasticity.

## 2. Materials and Methods

### 2.1. Field Surveys and Species Identification

Field surveys were carried out between 2014 and 2025 at 144 sampling sites distributed across Morocco. The sites were selected to represent a broad spectrum of environmental conditions and aquatic habitat types, including estuaries, streams, irrigation canals, lagoons, ponds, lakes, and brackish marshes. Macroinvertebrate communities were surveyed with particular attention to the invasive leech *H. europaea*. Specimens were sampled from a variety of microhabitats within each site using sweep nets, dip nets, forceps, and a Surber sampler (20 × 25 cm). At each station, eight Surber subsamples were collected, corresponding to a total sampled area of 0.40 m^2^. The abundance of *H. europaea* was standardized and expressed as density (number of individuals per 0.40 m^2^). A comprehensive list of sampling sites, together with the corresponding densities of *H. europaea*, is provided in the Supplementary Materials (Table S1).

**Figure 1:**
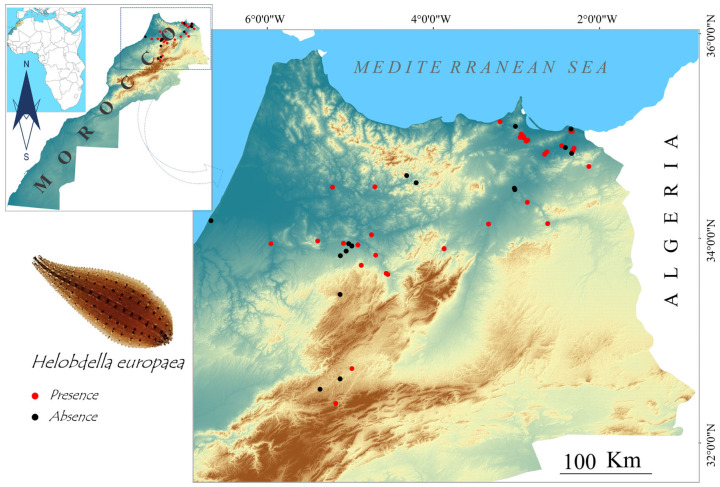
Habitus and updated map of *H. europaea* distribution in the continental waters of Morocco.

Specimens obtained during the historical sampling campaigns (2014–2018), as well as those from recent surveys (2019–2025), were preserved in 70% ethanol and subjected to detailed morphological analysis. The taxonomic identification relied on key diagnostic characters, including eyespot layout and nuchal scute configuration, in strict alignment with the morphological criteria used in our pioneering record of the species [14,15,16,22]. Indeed, *H. europaea* can be readily identified by the presence of five distinct rows of black-tipped papillae on the dorsal surface. Another key diagnostic feature is the dorsal pigmentation, which consists of numerous longitudinal dark grey stripes (Figure 1). In living specimens, the body reaches a maximum length of 10–20 mm and a width of 4–6 mm. The body is ovate–lanceolate and moderately dorsoventrally flattened, with the posterior region markedly broader than the tapering anterior (cephalic) end. The dorsum is fawn-colored, characterized by alternating longitudinal light and dark grey lines and stripes. Among these, the two median lines are the most prominent and closely spaced, extending from segments II–III to the posterior sucker. In contrast, submarginal and marginal lines are less distinct. White spots regularly interrupt the longitudinal stripes, often giving the median lines a chain-like appearance. No nuchal scute is present. The ventral surface is unpigmented and lacks any markings. The anterior region bears a pair of well-separated eyes, ranging in shape from punctiform to triangular. The posterior sucker is circular, with a diameter approximately equal to half of the body’s maximum width; it is flat and oriented ventrally. The species possesses a long, protrusible proboscis. The male and female gonopores are separated by a single annulus.

### 2.2. Environmental Data

The environmental variables measured at the sampled sites are presented in Table 1. In situ measurements of pH, electrical conductivity, and dissolved oxygen concentration were performed using a portable multiparameter probe (MPP350, WTW, Weilheim in Oberbayern, Germany).Water temperature was measured with a mercury thermometer (±0.1 °C accuracy), whereas flow velocity was estimated by recording the travel time of a floating cork stopper over a minimum distance of 1 m. Site elevation was determined using a handheld GPS receiver (eTrex 10, Garmin, Olathe, KS, USA). Physicochemical analyses were conducted exclusively at sites where *H. europaea* was recorded. At each sampling station, duplicate water samples were collected in 500 mL polyethylene bottles. Samples were preserved by adding 2 mL of concentrated hydrochloric acid to achieve a pH of approximately 2 and were transported to the laboratory in insulated coolers maintained at around 4 °C to minimize biological activity. Sampling, preservation, and transport procedures followed ISO standards 5667-6 [23], 5667-2 [24], and 5667-3 [25]. Laboratory analyses included the determination of sulfate (SO_4_^2−^), phosphate (PO_4_^3−^), nitrate (N–NO_3_^−^), and five-day biochemical oxygen demand (BOD_5_) concentrations. These analyses were performed in accordance with AFNOR standard methods [26] and the protocols described by Rodier et al. [27].

### 2.3. Statistical Analysis

To investigate the relationship between environmental factors and the presence of *H. europaea*, we first conducted a Principal Component Analysis (PCA) to uncover the multivariate environmental gradients separating colonized from uncolonized habitats. The ordinal variable for current velocity was converted to a numeric format (0, 1, 2) to be included alongside the continuous physicochemical parameters. The PCA was performed using the ade4, factoextra, and ggeffects packages. All 11 monitored environmental variables were retained in the exploratory PCA to provide a transparent, comprehensive representation of the full multivariate habitat matrix without introducing selection bias. Subsequently, to compare individual physicochemical parameters between sites harboring the species (presence) and those where it was absent, univariate statistical analyses were performed. We used ANOVAs followed by Tukey’s tests if conditions of normality and/or homoscedasticity were satisfied; otherwise, we used the non-parametric Kruskal–Wallis test followed by Wilcoxon tests. The resulting differences and significance levels were visualized via boxplots using the ggplot2 package. A total of 144 sites were surveyed, of which 49 were retained for subsequent analyses based on the availability of a complete and homogeneous set of environmental variables. *Helobdella europaea* was recorded at 32 of these sites. Other surveyed sites where the species was absent were not included in the following analyses because some environmental parameters were missing. All statistical analyses and data visualizations were performed using R software (version 4.1.3).

## 3. Results

*Helobdella europaea* appears to be widely distributed throughout the northern region of Morocco (Figure 1). The species was recorded at 32 of the 144 surveyed sites, occurring across a broad spectrum of freshwater habitats. These include high-altitude lakes (e.g., Lake Zerrouka), permanent springs, potamal sections of large rivers (e.g., the Moulouya River), and anthropogenic environments such as artificial canals in Berkan and Selouan. At the surveyed locations, *H. europaea* was typically associated with shallow, stagnant, or slow-flowing waters, where it was frequently observed among algal mats and macrophytes, beneath large stones, or attached to artificial substrates such as plastic debris. Future surveys could reveal other populations of this species and extend its known range in Morocco and North Africa.

The invasive leech *H. europaea* exhibited a high degree of ecological plasticity with respect to water physicochemical characteristics and abiotic environmental conditions across the surveyed localities, as reflected by its abundance patterns (Table S1, Supplementary Materials). In particular, the species was recorded across a broad range of electrical conductivity values, occurring in weakly mineralized waters with a minimum conductivity of 250 μS·cm^−1^ at Aval Cherraa River, as well as in highly mineralized waters, where conductivity reached 2469 μS·cm^−1^ at Barrage Za. Moreover, *H. europaea* also demonstrated tolerance to substantial spatiotemporal variation in dissolved oxygen concentrations. The species was observed in highly oxygenated waters, with concentrations up to 12.0 mg·L^−1^ at Dardoura River, and in relatively poorly oxygenated environments, with a minimum concentration of 5.6 mg·L^−1^ recorded at Melloulou River. Furthermore, the species exhibited a broad tolerance to water quality conditions associated with organic pollution. It was found in habitats characterized by nitrate concentrations ranging from 0.08 to 21.4 mg·L^−1^, sulfate concentrations from 25 to 279 mg·L^−1^, phosphate concentrations from 0.0 to 1.4 mg·L^−1^, and BOD_5_ values between 1.05 and 13.0 mg·L^−1^. Throughout the study period, *H. europaea* occurred predominantly in waters with near-neutral to slightly alkaline pH values (6.5–8.8) and across a wide thermal range (14–29 °C). The species was recorded at sites with water depths varying from 10 to 55 cm, further highlighting its ecological versatility.

**Figure 2:**
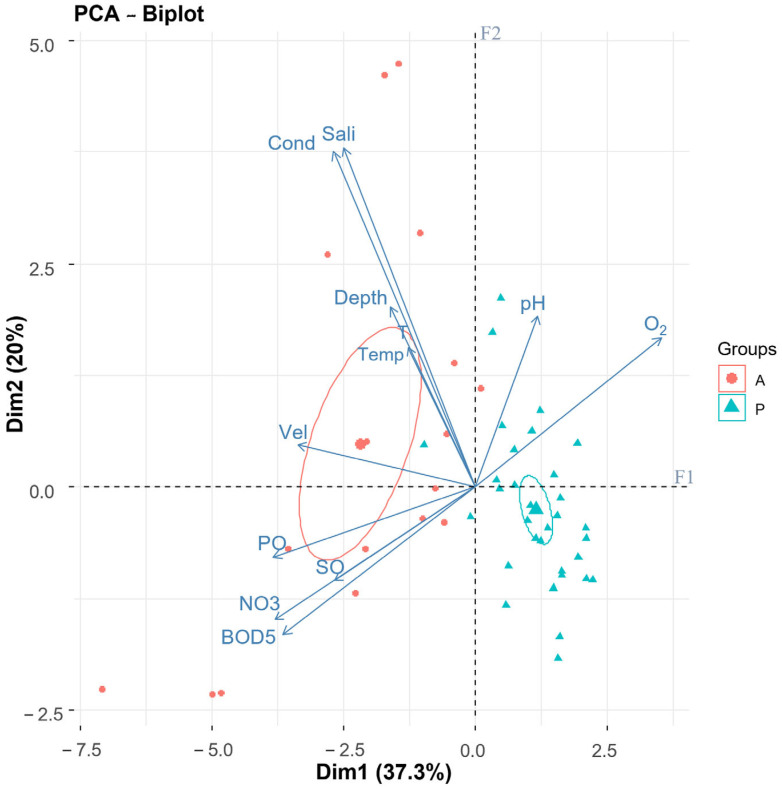
Principal Component Analysis (PCA) biplot showing the relationships between environmental variables and sampling sites with presence (blue triangles: P) and absence (red circles: A) of *H. europaea*. The vectors represent the measured environmental variables, including conductivity (Cond), salinity (Sali), water depth (Depth), flow velocity (Vel), pH, dissolved oxygen (O_2_), nitrate concentration (NO_3_), biochemical oxygen demand (BOD_5_), phosphate concentration (PO), and sulfate concentration (SO).

Figure 2 presents a Principal Component Analysis (PCA) biplot of water quality variables, illustrating the relationships between physicochemical parameters (blue vectors) and the distribution of sampling sites where *H. europaea* was either absent (A, red symbols) or present (P, blue symbols). The first principal component (Dim1, F1), explaining 37.3% of the total variance, represents a pollution gradient that increases from right to left. The second principal component (Dim2, F2), accounting for 20.0% of the total variance, reflects a salinity gradient that increases from bottom to top. The 95% confidence ellipses clearly separate sites with species presence from those where the species was absent, indicating distinct environmental conditions associated with the occurrence of *H. europaea*. Sites where the species was absent were primarily associated with higher salinity, greater water depth, and increased flow velocity. In contrast, sites supporting *H. europaea* tended to be characterized by higher dissolved oxygen concentrations and moderately alkaline pH values.

**Figure 3:**
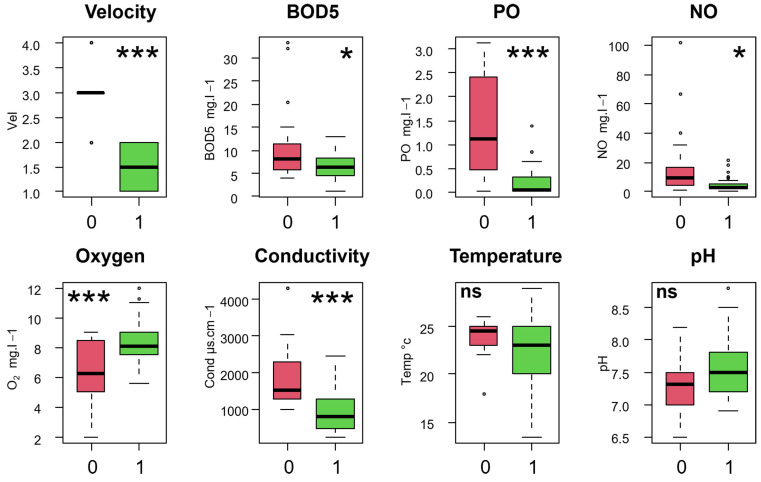
Boxplots of environmental variables at sampling sites with (1) and without (0) the invasive leech *H. europaea* in Morocco. Statistical significance is indicated by asterisks (***: *p* < 0.001, *: *p* < 0.05, ns: non-significant). Abbreviations for environmental variables are as follows: Vel = current velocity, BOD_5_ = biochemical oxygen demand, PO = phosphate concentration, SO = sulfate concentration, DO = dissolved oxygen, Cond = electrical conductivity, Temp = water temperature, and pH = potential of hydrogen.

Environmental parameter comparisons between colonized (presence, 1) and uncolonized (absence, 0) sampling sites revealed that the distribution of the invasive leech *H. europaea* (Figure 3) is highly significantly influenced by water velocity (*p* < 0.001, ***) and dissolved oxygen (*p* < 0.001, ***), showing a distinct preference for slow-flowing, well-oxygenated microhabitats. Regarding water chemistry, the presence of *H. europaea* was highly significantly associated with lower electrical conductivity (*p* < 0.001, ***) and reduced orthophosphate levels (*p* < 0.001, ***), while both biochemical oxygen demand (BOD_5: *p* < 0.05, *) and nitrogen compounds (NO: *p* < 0.05, *) were also significantly lower. Taken together, these results demonstrate that the species preferentially colonizes environments with lower nutrient enrichment and reduced organic pollution loads. Conversely, no statistically significant differences were observed for water temperature (*p* ≥ 0.05, ns) and pH (*p* ≥ 0.05, ns), highlighting a high tolerance and broad ecological plasticity of *H. europaea* toward these two parameters within the monitored ecosystems.

## 4. Discussion

*Helobdella europaea* is a globally successful invasive species with a complex taxonomic history spanning the past four decades. Initially described as *Helobdella striata* (Kutschera, 1985) in Germany and long considered the second native European *Helobdella* species [16], molecular and historical evidence later confirmed its North American origin and subsequent multi-route global introductions. To date, its non-native distribution has encompassed an extensive range, including Australia, New Zealand, South Africa, Hawaii, Fiji, California, the Netherlands, Taiwan, Spain, Hungary, Ukraine, Portugal, and Morocco [14,17,18,22,28,29,30,31,32,33,34,35,36].

In Morocco, the first confirmed record of *H. europaea* was reported in 2019, based on the identification of three preserved specimens collected in 2014 from the upper Moulouya and Selouane ditch systems [22]. While the precise introduction pathway remains unverified, it most likely mirrors that of other non-native aquatic taxa in the region: the international aquarium trade of exotic plants and animals, followed by accidental or intentional release into natural freshwater systems [21]. However, given the historically limited research on Moroccan Hirudinea and potential morphological misidentifications with native species such as *Placobdella costata* (Fr. Müller, 1846), *H. europaea* may have been cryptically present long before its formal detection. Our current findings document the widespread occurrence of this invasive leech across Moroccan hydrosystems and provide evidence of its successful establishment in multiple freshwater habitats.

The invasive success of *H. europaea* is inherently linked to its high ecological plasticity and effective passive dispersal mechanisms, such as hitchhiking on aquatic macrophytes or waterfowl [17,34,35]. Our statistical results refine the ecological niche of the species in North Africa. The highly significant preference for low water velocity and well-oxygenated waters confirms that the species thrives in stable, lentic microhabitats. Regarding vertical distribution, *H. europaea* demonstrated remarkable plasticity, establishing populations across a wide altitudinal gradient from lowland coastal anthropogenic systems to high-altitude lakes in the Middle Atlas. This suggests that the species is not constrained by altitude itself, but rather by local microhabitat stability, particularly low water velocity and sufficient oxygenation. Furthermore, its significant correlation with lower organic loads and reduced nutrients indicates that while *H. europaea* is highly plastic, its initial establishment is favored by relatively well-preserved or moderately enriched river sections rather than heavily degraded waters. Conversely, the non-significant effect of temperature and pH underscores a broad physiological tolerance, a critical adaptive trait for surviving the sharp seasonal fluctuations and hydrological stress typical of Moroccan freshwater ecosystems.

This ecological flexibility is coupled with an opportunistic, generalist feeding strategy. *Helobdella europaea* acts as both a predator and a scavenger, consuming a broad spectrum of macroinvertebrates—including oligochaetes, insect larvae, crustaceans, and gastropods [37]—as well as dead vertebrates like fish and amphibian larvae [37]. Recent reports even suggest potential facultative parasitism through attachment to freshwater turtles [34,38]. In Mediterranean freshwater ecosystems, such trophic generalism poses a severe ecological risk through competitive exclusion or direct predation on native fauna unadapted to such opportunistic invaders [14]. In northern Morocco, this threat is particularly critical within the Moulouya and Sebou river basins, major hotspots of freshwater biodiversity and endemism that harbor numerous micro-gastropods with highly restricted ranges, particularly crenobiont spring snails of the family Hydrobiidae, e.g., *Aghbalia aghbalensis* Glöer, Mabrouki & Taybi, 2020; *Ainiella zahredini* Taybi, Glöer & Mabrouki, 2022; *Idrisiella bourkaizensis* Mabrouki, Glöer & Taybi, 2022; *Ifrania bahhouensis* Mabrouki, Glöer & Taybi, 2022; and *I. zerroukansis* Glöer, Mabrouki & Taybi, 2020, which are exceptionally vulnerable to direct predation by this invasive leech [39]. Alarmingly, the Sebou and Moulouya basins are increasingly acting as hubs for cumulative biological invasions. The rapid spread of *H. europaea* occurs alongside the introduction of other non-native freshwater invertebrates [21,40], including the Asian predatory leech *Barbronia weberi* (Blanchard, 1897) [41]. The co-occurrence of these exotic species warrants close attention and may pose a risk of ‘invasional meltdown’ processes, where non-native taxa could mutually facilitate each other’s establishment, potentially leading to the restructuring of entire benthic communities and the degradation of ecosystem services [21].

Looking forward, climate change may represent an additional factor influencing the future distribution dynamics of *H. europaea* in Moroccan and North African freshwater ecosystems. Climate projections for the Mediterranean region indicate increasing temperatures, more frequent drought events, and reductions in river discharge [42,43]. Although climate variables were not directly incorporated into the present study, our results provide ecological insights into how future environmental changes may affect this invasive species [40]. Specifically, the observed tolerance to a broad range of water temperatures, together with the significant association of *H. europaea* with low-water-velocity habitats, suggests that this species may benefit from the expansion of lentic or slow-flowing habitats resulting from hydrological alterations. The spatial distribution of our dataset highlights the critical role of river basin interconnections. In Morocco, *H. europaea* is heavily favored by modern anthropogenic infrastructures, such as inter-basin water transfer projects and extensive concrete irrigation canals. These artificial structures act as perennial ecological corridors, bypassing natural geographic barriers and allowing the species to spread across previously isolated streams and catchments. Additionally, passive overland dispersal via zoochory (attached to waterfowl) or human-mediated transport cannot be ruled out and likely explains the colonization of isolated reservoirs.

Furthermore, although *H. europaea* showed a strong association with highly oxygenated environments, its frequent occurrence in areas with dense macrophyte development and anthropogenically modified habitats, such as irrigation canals and reservoirs, may provide suitable refugia under changing environmental conditions. Specifically, intense daytime photosynthesis by aquatic plants can cause substantial dissolved oxygen supersaturation (>10 mg·L^−1^) even in stagnant or slow-flowing waters. This mechanism explains how low-flow conditions and high dissolved oxygen levels can coexist in these ecosystems [27,44]. Consequently, although dissolved oxygen is statistically significant in our models, its strict ecological limiting effect is likely weaker than that of flow velocity and salinity, as *H. europaea* exhibits a broad physiological tolerance (5.6–12.0 mg·L^−1^) to oxygen fluctuations. The potential role of climate-driven habitat modifications in promoting the spread of *H. europaea* remains a hypothesis that requires further investigation through the integration of long-term hydrological data, drought indices, and climate projections. Considering the increasing pressure on freshwater ecosystems in North Africa, the implementation of early detection protocols, long-term biomonitoring programs, and targeted ecological studies will be essential to better predict and manage the potential impacts of this invasive species on native freshwater biodiversity.

One limitation of the present study concerns the taxonomic identification of *H. europaea*. This species belongs to the *Helobdella triserialis* (Em. Blanchard, 1849) species complex, a group in which species delimitation based solely on morphological characters is known to be challenging due to the high degree of morphological similarity and phenotypic variability among taxa [45]. Consequently, although the specimens examined were identified as *H. europaea* based on the currently available morphological diagnostic criteria, a certain degree of taxonomic uncertainty cannot be completely excluded in the absence of molecular confirmation [46]. Nevertheless, this limitation does not affect the main ecological findings of the present study, which focus on habitat preferences, environmental associations, and the invasive occurrence of this taxon in Moroccan freshwater ecosystems. Future research should integrate molecular approaches, such as DNA barcoding and phylogenetic analyses, in combination with morphological examination to confirm species identity, resolve taxonomic ambiguities within the *H. triserialis* complex, and improve our understanding of the distribution, invasion dynamics, and ecological impacts of *Helobdella* species in North Africa.

## 5. Conclusions

Our study demonstrates that *H. europaea* is established across northern Moroccan freshwater ecosystems. Although its first confirmed record was reported in 2019 based on specimens collected in 2014, the current findings reveal its occurrence across multiple freshwater habitats in Morocco. The species exhibits remarkable ecological flexibility, allowing it to colonize a wide range of habitats while tolerating substantial environmental variation. The spread of *H. europaea* raises significant conservation concerns, particularly in biodiversity-rich basins such as the Sebou and Moulouya, where numerous endemic freshwater species occur. Its opportunistic feeding behavior, combined with the increasing accumulation of other non-native aquatic organisms, warrants close attention as it may pose a risk of ecological pressure on native communities and potential ecosystem restructuring. Furthermore, while it remains a hypothesis that requires direct long-term data verification, projected climate-driven hydrological alterations, including reduced river flows and the expansion of lentic habitats, could potentially create increasingly favorable conditions for its continued spread across Morocco and throughout North Africa. Given these findings, *H. europaea* should be considered an emerging component of Morocco’s invasive freshwater fauna. Strengthening surveillance programs, implementing long-term biomonitoring schemes, and improving our understanding of its ecological interactions and hydrological dispersal pathways will be essential for anticipating future invasions and safeguarding the region’s unique freshwater biodiversity.

## Figures and Tables

**Table 1:** Environmental variables measured at sampled sites.

Environmental Variables	Units	Code
pH		pH
temperature	°C	Temp
Conductivity	µs·cm^−1^	Cond
Dissolved oxygen concentration	mg·L^−1^	O_2_
Ammonium	mg·L^−1^	NH
Sulphates	mg·L^−1^	SO
Orthophosphate	mg·L^−1^	PO
Biological oxygen demand	mg·L^−1^	DBO_5_
Depth	m	Dep
Current velocity (ordinal variable)	[0 (0–15), 1 (15–50), 2 (>50)] cm·s^−1^	Vel

## Data Availability

The raw data supporting the conclusions of this article will be made available by the first author upon request.

## Supplementary Materials

The following supporting information can be downloaded at https://www.mdpi.com/article/10.3390/biology15151228/s1, Table S1: Spatiotemporal variations in physicochemical water parameters and abiotic factors across the studied sampling stations. With conductivity (Cond), salinity (Sali), water depth (Depth), flow velocity (Vel), pH, dissolved oxygen (O_2_), nitrate concentration (NO_3_), biochemical oxygen demand (BOD_5_), phosphate concentration (PO), sulfate concentration (SO), density (number of individuals per 0.40 m^2^), presence (P), and absence (A).
