# Supplementary material for: Range Expansion and Environmental Determinants of the Invasive Freshwater Leech Helobdella europaea (Hirudinea: Glossiphoniidae) in Morocco
Source: Biology (Basel). 2026 Jul 23;15(15):1228. doi: 10.3390/biology15151228 (PMC13465163; doi:10.3390/biology15151228)
Supplement: Supplementary file 1 [file biology-15-01228-s001.zip › biology-4412138-supplementary.pdf]

Table S1. Spatiotemporal variations in physicochemical water parameters and abiotic factors across the studied sampling stations.

Code	Locality	Lat_Y	Long_X	Sampling Dates	Vel	Depth	pH	Temp	Sal	Cond	O2	NO	SO	PO	BOD5	Density	Presence
M1	Ait Boulmane	32,61572	-5,33033	03/05/2014	3	35	7,1	25	1,5	2291	7,3	1,3	52	0,02	5,93	0	A
M5	Boumia	32,71761	-5,09797	14/06/2014	4	50	7,3	25,2	1,1	1700	6,1	4,1	170	1,59	6,9	0	A
M6	Zaïda	32,81750	-4,95917	14/06/2014	1	35	7,1	20	0,4	605	7,2	2,3	55	0,01	5,89	2	P
M8	Aval Anzar Oufounas	32,47824	-5,14817	02/05/2014	2	45	7,2	18	0,3	370	9,1	0,1	50	0,02	1,05	15	P
M18	Moulouya av Za	34,56141	-3,03049	29/07/2018	3	45	7,4	25	0,8	1220	6,2	17	342	1,05	8	0	A
M19	Sebra	34,88639	-2,66250	15/10/2017	2	50	7,4	20	0,8	1094	8,9	5,4	279	0,5	4,93	6	P
M20	Safsaf	34,90765	-2,63579	22/10/2017	2	40	7,5	23	0,8	1194	7,9	2,9	227	0,02	1,49	6	P
M25	Moulouya Mouth II	35,12392	-2,34121	23/10/2017	2	70	7,5	25	2,8	4308	8,5	9,4	35	0,46	4,4	0	A
S2	Amont O El Bared	33,98306	-3,87106	08/06/2014	2	25	7,1	14	0,3	341	8,5	9,5	35	0,56	5	1	P
S12	Aval Melloulou	34,21699	-3,34456	08/07/2018	2	5	7,6	26	0,6	936	5,6	7,4	65	0,05	6,8	1	P
Z9	Barrage Za	34,20642	-2,64786	22/07/2018	1	40	7,3	25	1,6	2469	8	3	69	0,02	5,2	3	P
Z10	Amont Taourirt	34,42100	-2,88136	22/07/18	2	50	7,3	25	0,9	1366	7,4	2,2	51	0,02	4,2	4	P
Z11	Melg el Ouidane	34,54625	-3,02531	29/07/2018	3	50	6,9	24	0,9	1392	5,2	2,8	52	0,03	10,2	0	A
O2	Aval Zeghzel	34,88564	-2,34280	15/10/2017	4	40	7,3	23,5	1,3	1970	3,2	101	225	2,54	33,2	0	A
O3	Cherraa _Avant STEP	34,94544	-2,41347	15/10/2017	2	50	6,8	22	1	1485	5	6	129	1,11	5,75	0	A
O4	Aval Cherraa	34,96431	-2,45719	15/10/2017	1	35	7,5	16,9	0,2	250	7	1,2	40	0,63	7,4	22	P
O5	Cherarba	35,10519	-2,34583	30/07/2018	1	50	7,4	13,5	0,4	475	8,1	2,9	55	0,01	1,81	3	P
O6	canal Saïdia	35,09972	-2,32836	22/02/2016	1	20	7,2	23	0,3	435	8,2	1,7	35	0,01	1,3	2	P
O10	Ain Sfa	34,75342	-2,14333	15/06/2015	1	40	7,2	24	0,2	326	8,1	4,3	35	0,02	6,2	8	P
O13	Canal Berkane	34,93522	-2,31142	29/07/2014	1	25	8,2	25	0,9	1425	9	21	70	0,3	9	16	P
O15	Waoullout	34,91123	-2,32896	07/02/2021	1	20	7,5	20	0,6	779	7,5	3,4	62	0,02	6,14	7	P
N1	Oued Selouan	35,05920	-2,94878	17/08/2018	2	43	7,5	19,2	0,3	375	6,5	9,5	40	0,84	7,54	8	P
N4	Dardoura	35,05306	-2,90525	12/08/2018	2	30	8,1	24	0,5	818	12	2,4	53	0,2	6	11	P
N5	O Messoussate	35,06350	-2,90639	12/08/2018	2	0	8,1	25	0,6	1006	11	2,3	78	0,32	10	5	P
N6	Saguia Selouane	35,08731	-2,93439	12/08/2018	2	10	7,4	25	1	1478	9	10	65	0,04	8	46	P
N7	Barrage Arabat	35,01775	-2,87667	13/08/2018	1	30	8,1	25	0,9	1475	10	3,3	62	0,32	9,3	20	P
N11	Oued Kert	35,21358	-3,18372	30/08/2018	2	48	7,7	19	0,4	536	8	4,7	38	0,35	6,68	3	P
N13	Afluent bar Oul	35,02838	-2,85897	13/08/2018	2	30	7,5	25	1,3	1945	8	18	50	1,4	13	13	P
P1	Oued Issekkajen	35,16492	-3,00178	10/09/2023	3	60	7,6	25	1,8	2870	9	9,8	90	0,4	5,7	0	A
F1	Oued El Menzel	34,03996	-4,99268	04/07/2021	2	45	7	23	0,8	1230	5	15	90	3,04	15	0	A
F2	Barrage El-Guaâ	34,01811	-4,95494	04/07/2021	3	50	6,5	24	0,9	1370	2,2	40	89	2,4	32	0	A
F3	Lake Zerrouka	33,54360	-5,09466	14/02/2020	3	60	7,5	25	2,8	4308	8,5	9,4	35	0,46	4,4	0	A
F4	lake Sidi Bougha	34,25222	-6,66664	21/04/2016	3	70	8,2	18	2,3	3034	6,5	8,7	48	3,12	11,4	0	A
F5	Lake sidi Chahed	34,06746	-5,35661	09/05/2021	1	45	8,8	23	1,5	2240	7,1	13	68	0,03	5,02	4	P
F12	Oued Fes S1	34,04251	-5,05321	02/06/2021	2	30	7	21	0,5	647	11	2,5	25	0,01	2,5	2	P
F18	Ain Chkef (1)	33,96806	-5,02289	06/12/2019	3	40	6,7	23	1,1	1572	2	67	209	2,1	20,3	0	A
F21	Ain Chkef (4)	33,96806	-5,02289	26/12/2019	4	35	7,6	25	1	1523	6	32	261	2,82	11,2	0	A
F22	Barrage Allal El	33,92693	-4,67543	01/04/2021	1	40	7,8	24	0,5	700	7,9	2,7	45	0,85	8,92	13	P
F23	Barrage Al Wah	34,59280	-5,18042	21/07/2020	1	30	7,8	25	0,4	679	8,1	4,5	41	0,18	9,3	1	P
F24	Oued Bourkaiz	33,92271	-5,08893	09/12/2020	3	40	7,4	24,5	0,6	981	7	15	98	0,9	7	0	A
F28	Barrage El Ganzr	34,03848	-5,90548	20/07/2021	1	40	7,6	25,3	0,5	834	7,5	3,6	43	0,2	8,4	1	P
F30	Oued Aggaï	33,82844	-4,84533	06/06/2023	1	50	7	23	0,5	690	9,5	3,5	40	0,01	6	4	P
F32	Barrage Idris 1er	34,12683	-4,72342	21/10/2023	1	42	8,2	21	0,6	923	8,6	1,9	69	0,07	8,5	5	P
F33	sources oued se	33,73683	-4,53331	30/05/2022	2	40	7,2	20	0,3	413	10	1,1	31	0,01	2,5	1	P
F34	confluence Ain	33,74794	-4,55478	30/05/2022	2	55	7	21	0,3	468	8,5	2,7	54	0,01	4,12	3	P
F37	Ghadir hama	34,70203	-4,30336	13/07/2024	4	65	7,2	23	0,9	1275	9	1,4	27	0,01	3,93	0	A
F38	Aval Assfalou	34,63000	-4,19072	13/07/2024	3	50	8	26	0,7	1143	8,5	1,5	62	1,63	10,5	0	A

F40	Barrage Sahla	34,59425	-4,67967	13/07/2024	2	40	8,5	25	0,6	950	8	2,5	50	0,05	7,5	4	P
F42	Oued Sidi Hraze	34,02764	-4,88440	10/10/2023	1	20	6,9	29	0,9	1564	6	2,5	23	0,01	8,45	14	P
